# Time Points: A Gestural Study of the Development of Space–Time Mappings

**DOI:** 10.1111/cogs.12801

**Published:** 2019-12-09

**Authors:** Patrick Burns, Teresa McCormack, Agnieszka J. Jaroslawska, Patrick A. O'Connor, Eugene M. Caruso

**Affiliations:** ^1^ School of Psychology Queen's University Belfast; ^2^ Anderson School of Management University of California, Los Angeles

**Keywords:** Space, Time mapping, Gesture, Embodied cognition, Child development

## Abstract

Human languages typically employ a variety of spatial metaphors for time (e.g., “I'm looking forward to the weekend”). The metaphorical grounding of time in space is also evident in gesture. The gestures that are performed when talking about time bolster the view that people sometimes think about regions of time as if they were locations in space. However, almost nothing is known about the development of metaphorical gestures for time, despite keen interest in the origins of space–time metaphors. In this study, we examined the gestures that English‐speaking 6‐to‐7‐year‐olds, 9‐to‐11‐year‐olds, 13‐to‐15‐year‐olds, and adults produced when talking about time. Participants were asked to explain the difference between pairs of temporal adverbs (e.g., “tomorrow” versus “yesterday”) and to use their hands while doing so. There was a gradual increase across age groups in the propensity to produce spatial metaphorical gestures when talking about time. However, even a substantial majority of 6‐to‐7‐year‐old children produced a spatial gesture on at least one occasion. Overall, participants produced fewer gestures in the sagittal (front‐back) axis than in the lateral (left‐right) axis, and this was particularly true for the youngest children and adolescents. Gestures that were incongruent with the prevailing norms of space–time mappings among English speakers (leftward and backward for past; rightward and forward for future) gradually decreased with increasing age. This was true for both the lateral and sagittal axis. This study highlights the importance of metaphoricity in children's understanding of time. It also suggests that, by 6 to 7 years of age, culturally determined representations of time have a strong influence on children's spatial metaphorical gestures.

## 1 Introduction

When people talk about time, they often employ spatial metaphors (see Bender & Beller, 2014, for a review). For example, in English, future events are often described as being “ahead of us,” “just around the corner,” or “on the horizon.” In each of these examples, an event occurring at a future time period is referred to as if it were a location in space. Though on occasion space may be described in terms of time (e.g., “I live 5 min from the station”)*,* spatial metaphors for time appear to be much more common (Haspelmath, 1997). What does the metaphorical mapping of time onto space reveal about our grasp of the concept of time? In one of the seminal works to address this question, George Lakoff and Mark Johnson argued that our conceptual system is largely metaphorical in nature and that this is revealed in the way we speak about abstract concepts, including time (Lakoff & Johnson, 1980). Lakoff and Johnson noted that concrete tangible domains are often used as source domains for the metaphoric structuring of more abstract intangible concepts. They argued that spatial metaphors for time are not just a matter of surface language use; rather, thinking about time is inherently metaphorical in nature and involves spatial representations. In this study, we investigated the developmental origins of space–time mappings in order to assess the claim that thinking about time is inherently metaphorical.

Underlying the metaphorical mapping of time onto space are a number of different (and sometimes competing) models of the relationship. For example, in the *moving ego* model the speaker is moving through time encountering fixed events along the way, for example, “we're coming up to our anniversary.” In the *moving time* model the ego is static and it is events in time that approach and then recede (e.g., “Monday's deadline is fast approaching”; Clark, 1973). Both moving ego and moving time metaphors can take the ego as the referent point, and generally the location of the ego coincides with the deictic “now.” Time referent models are also found, whereby events are described as locations relative to one another, for example, “Tuesday comes after Monday” (Núñez & Cooperrider, 2013). Languages can employ more than one model and individuals seem to switch from one to other with relative ease (Moore, 2006; Núñez, Motz, & Teuscher, 2006). Although the *time as space* metaphor appears to be universal (Evans, 2004, though, controversially, see Sinha, Sinha, Zinken, & Sampaio, 2011), the way in which time is mapped onto space varies across speech communities (e.g., Bender & Beller, 2014; Boroditsky, 2001; Boroditsky & Gaby, 2010; Fuhrman & Boroditsky, 2010). In English, as with many other European languages, the sagittal bodily axis is often the ground for the metaphorical mapping of time onto space, with the past connate with the space behind the body and the future the space in front (Bender & Beller, 2014; Núñez & Cooperrider, 2013). In other languages, the mapping is reversed. Núñez and Sweetser (2006) describe the Aymara language, spoken in the Andean highlands of western Bolivia, which uses the same word for *front* as for *past* and the same word for *back* as for *future*. In Mandarin, sagittal metaphors for time are employed though unusually examples of both *past‐in‐front*/*future‐behind* metaphors and *past‐behind*/*future‐in‐front* metaphors are found (Gu, Zheng, & Swerts, 2018). Mandarin is also notable for the availability of vertical metaphors for time (*past* is *up* and *future* is *down*) as in the examples 上個月/shàng gè yuè, meaning last month [lit. “above month”] and 下個月/xià gè yuè, meaning next month [lit. “below month”] (Boroditsky, 2001; Fuhrman et al., 2011; Miles, Tan, Noble, Lumsden, & Macrae, 2011).

### 1.1 Gestures and the spatialization of time

Evidence for a connection between time and space in thought is not only found in metaphoric language but is also seen in the gestures that accompany temporal talk. These gestures are typically interpreted as drawing on the source domain of space in much the same way that verbal metaphors do (Cienki & Müller, 2008). Indeed, the same cross‐linguistic differences concerning the way that people metaphorize time in speech are also evident in gesture. The Aymara, for example, gesture for the past as in front and the future behind (Núñez & Sweetser, 2006; see also Sullivan & Bui, 2016, for a similar pattern among Vietnamese). Núñez, Cooperrider, Doan, and Wassmann (2012) describe the Yupno of Papua New Guinea, a people who use topographic features of their environment to spatialize time. They live on a mountainside and when outdoors they point downhill when using deictic temporal phrases that refer to the past and uphill when using future terms (see also Fedden & Boroditsky, 2012, for a similar example of the Mianmin of Papua New Guinea). Geocentric frameworks for spatializing time have also been reported such as the example of the Australian Aborigine community of Pormpuraaw, who organize temporal sequences in a card arrangement task according to cardinal directions (east for earlier and west for later) and also gesture eastward for past events (Boroditsky & Gaby, 2010). There is also some evidence from studies of gesture to suggest that spatialization of time is not necessarily linear. Whereas the gestures produced in a variety of bodily axes by speakers of many languages appear to be based on a linear notion of time with complementary past and future directions, Le Guen and Pool Balam (2012) argued that Yucatec Maya speakers prioritize a cyclical notion of time, one in which events continually replace one another in a recurrent manner. Consistent with this, gestures that Yucatec Maya speakers produce suggest that time is spatialized in a way that is directionless: Distances in time seem to be spatialized radially from the body, with both the distant past and distant future marked in gesture in the same way (by pointing high above the speaker's body).

One issue raised by these studies is the relation between linguistic spatial metaphors and the spatialization of time manifest in gesture. There is some evidence to suggest that there is a close relation between the two, to the extent that the specific space–time metaphor currently being employed in a given language can influence gesture production: Gu, Mol, Hoetjes, and Swerts (2017) found that Mandarin speakers used more vertical gestures when discussing temporal terms that employ vertical spatial metaphors than compared with temporal terms that were neutral. However, studies that have analyzed the gestures English speakers make when talking about time indicate that their speech and gesture often come apart (Casasanto & Jasmin, 2012; Cooperrider & Núñez, 2009). English speakers in certain circumstances are more likely to gesture in the lateral plane (leftwards for the past and rightwards for the future) than in the sagittal plane when talking about time. What is striking about this is that, although there are a number of cultural conventions that assign a left‐to‐right mapping of earlier to later times (e.g., calendars, writing direction, graphs), English does not lexicalize time on to the lateral axis. Indeed, it seems that left–right linguistic metaphors for time do not occur in any language (Clark, 1973; Haspelmath, 1997; Radden, 2004; though for a recently reported exception, see Hendricks, Bergen, & Marghetis, 2018). Nevertheless, Cooperrider and Núñez reported that lateral gestures for temporal information by far exceed gestures in the sagittal plane in their study of American English speakers.

One possible reason why gestures in the lateral plane were so frequent in the Cooperrider and Núñez's (2009) study is that the stimuli depicted the history of the universe in pictorial form unfolding from left to right (participants had to describe the picture to an interlocutor seated directly opposite). The stimuli employed by Casasanto and Jasmin (2012), by contrast, were verbal. In one of their studies, participants (naïve to the purpose of the study) read short stories that described sequences of events, sometimes with spatial metaphors for time embedded and sometimes without, and then relayed the story to an interlocutor seated opposite. Despite the differences in Casasanto and Jasmin's procedure, their findings were similar to those of Cooperrider and Núñez, with participants producing many more lateral gestures than sagittal gestures; moreover, the inclusion of spatial metaphors had no effect on the axis used in participants' spontaneous gestures (cf. Gu et al., 2017). However, in a different study in the same paper, participants were explicitly asked to demonstrate how they would gesture to indicate past and future events: Gestures were as likely to be produced in the sagittal as the lateral plane. The authors argued that the explicit instructions to produce gestures resulted in the activation into conscious awareness of the sagittal mapping of time that appears in language.^1^ Interestingly, both the Cooperrider and Núñez study and that of Casasanto and Jasmin report individuals gesturing laterally while simultaneously using sagittal verbal metaphors to describe time. Walker and Cooperrider (2016) also report that, albeit in a minority of cases, people will combine gestures from both the sagittal and lateral planes, providing further evidence of co‐activation of two time–space metaphors.

### 1.2 Developmental predictions regarding time–space gestures

As has been described, a large number of cross‐cultural studies have revealed a great diversity in the way that human societies map time on to space, as evidenced by both linguistic metaphor use and gesture production. However, surprisingly little is yet known about the developmental emergence of spatial metaphors for time in any population, although behavioral studies have begun to examine English and Italian‐speaking children's lateralized representations associated with deictic time words (Nava, Rinaldi, Bulf, & Cassia, 2017; Tillman, Tulagan, Fukuda, & Barner, 2018). In this study, we explore the development of space–time mapping further by examining developmental change in the gestures that English‐speaking children produce when talking about time.

It is possible to frame two competing developmental predictions about the types of gestures we might expect to observe. First, one might predict that sagittal gestures would be particularly frequent in children. Lakoff and Johnson have argued that simple spatial concepts such as *front‐back* and *up‐down* ground much of our conceptual system. Orientational metaphors*,* as they refer to them, employ basic spatial concepts as the source domain for a wide range of abstract concepts. Consistent with this, Rinaldi and colleagues have argued that the sagittal mental time line directly arises from visual experience encountered through locomotion: as people move through their environments they see future events approach while past events are literally behind them (Rinaldi et al., 2016; Rinaldi, Vecchi, Fantino, Merabet, & Cattaneo, 2018). We might therefore expect that as soon as children start to think about time that they will begin to organize their temporal concepts on a sagittal mental time line, and therefore sagittal gestures might be dominant in children.

The alternative possibility is that sagittal gestures are not the dominant gesture type in children. The existence of linguistic communities in which the past in mapped to the front of the body and the future behind the body (Núñez & Sweetser, 2006) already suggests that sagittal representations of time are not necessarily grounded in sensorimotor experience in the way that Rinaldi and colleagues have argued (Rinaldi et al., 2016, 2018). Rather, such space–time mappings may depend on knowledge of the relevant cultural and linguistic metaphors with which children may be relatively unfamiliar. Indeed, even when children encounter the relevant metaphors in language, this may not straightforwardly lead to the acquisition of sagittal representations of time. Many expressions that are derived from a metaphorical or analogical mapping become fossilized over time (Leech, 1974), and even adults are not always alive to the metaphoric nature of some expressions (the so‐called dead or inactive metaphors; Bowdle & Gentner, 2005). Some spatial metaphors for time may be represented in the lexicon homophonically (Gentner, 2001), and it may only be later that children come to understand the correspondence between the two domains. Thus, it remains possible that children, in comparison with adolescents and adults, will produce relatively few sagittal gestures when speaking about time.

Does this mean that it is possible that children's gestures might in fact be more likely to be on the lateral plane? There has been considerable debate regarding the origins of space–time mappings on the lateral axis (e.g., Chatterjee, 2001; Dobel, Diesendruck, & Bolte, 2007; Tversky, Kugelmass, & Winter, 1991; Winter, Marghetis, & Matlock, 2015), but there is now general agreement that the *left‐past* and *right‐future* mapping seen in English‐speaking populations is linked to cultural conventions concerning the left‐to‐right spatialization of time (e.g., graphs, calendars, number lines etc., as well as writing direction; Núñez & Cooperrider, 2013). By comparison to adolescents and adults, children have much less experience of such conventions. Thus, there may be developmental increases in the extent to which gesturing in the lateral axis is observed, as children learn to internalize the left‐to‐right mapping of past to future (or earlier to later for non‐deictic terms).

However, we note that English‐speaking children in the early school years in the United Kingdom (where our study was conducted) will typically be receiving intensive tuition in reading and writing and about the number line that heavily emphasizes left‐to‐right representations of temporal sequences. Indeed, by 5 to 6 years of age, native English‐speaking children from North America prefer to arrange stickers representing events in a temporal sequence in a left‐to‐right order on a blank sheet of card, indicating acquisition of space–time mapping on a lateral plane (Tillman et al., 2018). This suggests that children in the early school years may, in principle at least, have already acquired a way of spatially representing time that could underpin gesture use. We now turn to considering whether there is other evidence that would suggest that children of this age could use such metaphors in their gestures.

### 1.3 The development of gesture

Children begin to gesture almost as soon as they begin to communicate (Goldin‐Meadow, 1999). At around 10 months of age, we see the emergence of deictic gestures, such as pointing, showing, and giving. These gestures are an important developmental milestone in their own right, presaging the emergence of speech. In the second year of life, gesture and speech operate relatively autonomously from one another, such that when children gesture they rarely speak and vice versa (Butcher & Goldin‐Meadow, 2000; Tellier, 2009). Gradually, however, we see increasing integration and synchrony between gesture and speech. Around 3 to 4 years of age, there is a marked increase in gesture, referred to as the gesture “explosion” (McNeill, 2015). The character of children's gesturing changes at this stage. In the early years, children's gestures predominantly involve pantomiming (“silent gestures”); however, from age 4 onwards, we see more adult forms of gesturing emerge, such as iconic gestures and beat gestures. McNeill identifies this age with the beginning of gesticulation, the process whereby gestures augment and dovetail with speech to enhance communication and understanding. Most significant for this study is the emergence of metaphoric gestures around 5 to 6 years of age (McNeill, 1992; Tellier, 2009). These gestures represent abstract content of one form or another, and typically embody common metaphors expressed in language. Take, for example, the conduit metaphor for communication, in which ideas are objects that can be transferred between individuals (Lakoff & Johnson, 1980; Reddy, 1979). McNeill describes gestures that embody the conduit metaphor, where children appear to hold an object in front, as if objectifying what they are saying (McNeill, 1992). That these and other metaphorical gestures begin to emerge by 5 to 6 years of age would suggest that children have the resources to produce metaphoric gestures for time by this age.

What of children's understanding of linguistic spatial metaphors for time? Around the same age, both Turkish‐ and English‐speaking children appear to understand at least some spatial metaphorical expressions for time (Özçalişkan, 2005; Stites & Özçalişkan, 2013), although it appears that the nature of the metaphor makes a difference. Stites and Özçalişkan found that both moving ego and moving time metaphors (metaphors in which the ego corresponds to the deictic “now”) were easier for children than time referent metaphors in which the temporal position of one event was described relative to another and independent of the perspective of an observer. Stites and Özçalişkan suggest that the early understanding of ego referent spatial metaphors reflects the importance of a first‐person perspective in the developing conception of time. Given that in English such metaphors involve space–time mapping on the sagittal axis, similarly to Rinaldi et al.'s (2016, 2018) sensorimotor hypothesis described above, this again raises the possibility that sagittal gestures will be particularly common in children relative to lateral gestures. In summary, it appears that by at least 6 years of age, children have the conceptual prerequisites to produce metaphoric gestures when discussing time. Nevertheless, given that there are further improvements in use and comprehension of conceptual metaphors throughout childhood (Vosniadou, 1987; Waggoner, Palermo, & Kirsh, 1997), it remains likely that the use of metaphoric gestures for time will increase developmentally across childhood and into adolescence.

## 2 The current study

In this study, we examined the gestures that children and adults made when asked to contrast the difference between a pair of temporal terms. To the best of our knowledge, there are only two previous studies that have examined children's gestures when talking about time. Iossifova and Marmolejo‐Ramos (2013) report that Bulgarian‐speaking 6‐ to 8‐year‐olds will point when cued to locations corresponding to past and future times; unfortunately, they do not report what axis children use nor do they examine developmental change (the focus of the study is a comparison between sighted and visually impaired children). Marghetis, Tillman, Srinivasen, and Barner (2014) provide some initial evidence that children as young as 5 years produce gestures in line with *past‐left* and *future‐right* mapping, but they do not report developmental profiles of gesture use nor examples of use of the sagittal plane. Thus, this study will provide the first detailed analysis of development of spatial gestures that map time periods on to regions of space.

We were particularly interested in (a) whether there were developmental changes in the likelihood that participants would use spatial gestures when talking about time, (b) whether these were increasingly likely to be canonical for an English‐speaking population in nature, (c) whether there were developmental changes in the plane on which gestures were located (lateral/sagittal), and (d) when contrasting two temporal terms that vary in the distance from the present, will this difference be marked in gesture. In addition to placing past and future terms on complementary axis directions, we take the marking of magnitude differences as further evidence that individuals are operating with a linear and extended mental timeline.

We adapted the task used by Marghetis et al. (2014) in which children were asked to explain the difference between pairs of temporal terms (e.g., “tomorrow” vs. “next week”). Unlike Marghetis et al. (2014), we explicitly asked participants to use their hands when explaining these differences. Although we acknowledge that explicit reminders to use one's hands comes with a potential cost to ecological validity, we felt that it was warranted given that initial pilot work established very low instance of gesture use in children without such instructions.

## 3 Method

### 3.1 Participants

There were 133 participants (70 females) recruited for this study across four age groups: thirty‐nine 6‐to‐7‐year‐olds (*M*_age_ = 84 months, range = 69–96 months, 22 females); thirty‐three 9‐to‐11‐year‐olds (*M*_age_ = 122 months, range = 108–144 months, 11 females); twenty‐one 13‐to‐15‐year‐olds (*M*_age_ = 174 months, range = 156–188 months, 11 females); and forty adults (*M*_age_ = 27 years 7 months, range = 18–62 years, 26 females). Children and teenagers were recruited via posters, flyers, and social media posts. Adult participants were recruited through notices across the home university of the lead author, and through a psychology research participation scheme. All of the children and adolescents tested were native English speakers. Four of the adult participants, although fluent in English, were non‐native English speakers. All analyses reported below include these participants as none of the analyses changed with their exclusion. All participants were tested in the developmental laboratory of the first author's host institution. Testing sessions were approximately 1 h 15 min in length and the gesture study participants completed a number of additional tasks concerning temporal and episodic cognition that are reported elsewhere (McCormack, Burns, O'Connor, Jaroslawska, & Caruso, 2019). All adult participants were paid £15 (UK pounds) for their participation in the testing session. Informed written consent for the study was obtained from adult participants and from the parents of child participants. In addition, children and adolescents provided written assent.

### 3.2 Materials and procedure

Participants sat on an office chair without any armrests during the testing session. An experimenter sat directly opposite them. Above the experimenter's head was a wall‐mounted camera which recorded the session. Participants were read aloud the following brief instructions: “I have different pairs of words for you and you have to tell me the difference between these words. I want you to tell me and use your hands to show me.” Participants were presented with six pairs of temporal terms: tomorrow versus yesterday; last week versus next week; this morning versus yesterday; tonight versus last year; tomorrow versus next week; last week versus next year. The six pairs were presented to participants in one of two counterbalanced orders. Within each pair, the order the two terms were introduced was kept constant. For each pair the experimenter asked the participant to explain the difference between the two terms. To ensure that the experimenter did not inadvertently perform any gestures during the task, he or she was instructed to hold with both hands a sheet of paper from which the task instructions were read aloud. If participants failed to gesture during the task, they were given verbal prompts by the experimenter reminding them to use their hands.

## 4 Analysis

Videos from each session were coded using ELAN video annotation software (https://tla.mpi.nl/tools/tla-tools/elan/). We adapted the gesture coding scheme outlined by McNeill (1992). Videos were first parsed such that co‐speech gestures produced with each of the 12 target temporal adverbs were identified and isolated. Any iconic gestures (i.e., those in which the gesture closely matched the semantic content of the speech) were removed from further analysis. In the present corpus, these almost exclusively consisted of pantomimed actions that depict activities that the participant performed (or intends to perform) at the time of reference (e.g., “tomorrow I will walk my dog”—pantomimes walking dog). From the remaining gestures, metaphoric spatial gestures were identified (i.e., gestures in which the source domain of space for time referents was represented in a hand movement). These mostly consisted of either finger points or arm extensions with either throwing motions, cutting movements of the hand, open palms, or the hands configured as if placing something to one side. Video recordings of these gestures were then clipped for coding. Where participants produced more than one spatial gesture when describing a given temporal adverb, the first gesture was the one that was clipped and coded. In focusing on the first gesture produced, our analysis follows that of the gesture elicitation studies of Casasanto and Jasmin (2012, Study 1) and Walker and Cooperrider (Study 1). Thus, in analyzing overall gesture rates, our unit of analysis is not total gestures produced but rather the presence/absence of a spatial gesture given the opportunity to explain a temporal term. This allowed us to examine age effects in the tendency to produce any spatial gesture when explaining a given temporal term; we note that in the majority of cases, participants produced a single gesture per term. Each identified gesture was coded along three dimensions: *orientation*, *direction,* and *congruency*. Orientation coded the bodily axis along which the gesture was produced (sagittal, lateral, or vertical), with an additional fourth category (origin) for gestures that were located around the origin of the three main axes just in front of the body's torso. Direction referred to the path of the gesture (rightward, leftward, forward, backward, upward, downward, center [i.e., origin gestures], hands moving together, hands moving apart). The subset of gestures that were produced in the lateral or sagittal planes were coded as either congruent or incongruent with canonical space–time mappings (leftward and backward for past and rightward and forward for future).

Of the six pairs of temporal adverbs selected, four pairs were asymmetrically distant from the present (e.g., tonight vs. last year). For these pairs, we additionally coded whether the asymmetry in temporal distance from the present was evident in the magnitude of the gestures, that is, in the extension of the gesture from the torso. The initial parsing of videos and coding of clips was done by one individual. A second rater coded a random selection of 30% of the clips. Interrater reliability between the two coders was high: orientation (*k* = .83, *p* < .01), direction (*k* = .85, *p* < .01), and congruency (*k* = .93, *p* < .01). For magnitude judgments, coders watched pairs of video clips without audio and judged whether the gestures were equivalent in size or whether one was bigger than the other. Interrater reliability for magnitude judgments was also high (*k* = .64, *p* < .01).

## 5 Results

In total, 58 iconic gestures (3.6% of observations) were identified and removed from subsequent analyses. Of these, 45 were produced by the 6‐to‐7‐year‐olds and the remaining 13 were produced by the 9‐to‐11‐year‐olds.

### 5.1 Proportion of spatial gestures by age group

We first examined the proportion of spatial gestures produced by each age group as a proportion of the total number of opportunities available (12 per participant). Spatial gestures were those that were coded as lateral, sagittal, or vertical in orientation. Overall, 72% of observations produced spatial gestures (see Table 2 for a breakdown by age group). Of the remaining observations, 7.8% produced origin gestures and 20.4% did not produce a gesture. The proportion of origin gestures did not vary by age group, Wald χ^2^(3) = 4.08, *p* = .25.

To account for the clustered nature of the data (observations were clustered by participant and by item), we analyzed these data with a series of multi‐level regression models fitted with a logit function to account for the binary nature of the dependent variable and fitted using the *lme4* package in *R* (Bates, Maechler, Bolker, & Waler, 2014; R Core Team, 2015). We first examined how the propensity to produce a spatial gesture varied by age group with the following model.

$$\mathrm{Pr}\left(spatial\;gesture\right)=logit^{-1}\left(\beta_{0}+Subj_{0j}+Item_{0k}+\beta_{1}.Age\;group+\epsilon_{i}\right)$$

$$Subj_{0j}∼N\left(0,\;\sigma_{\mathrm{subj}}^{2}\right),\;\text{for}\;j=1,\;\ldots ,\;132$$

$$Item_{0k}∼N\left(0,\;\sigma_{\mathrm{item}}^{2}\right),\;\text{for}\;k=1,\;\ldots ,\;8$$

**Figure 1:**
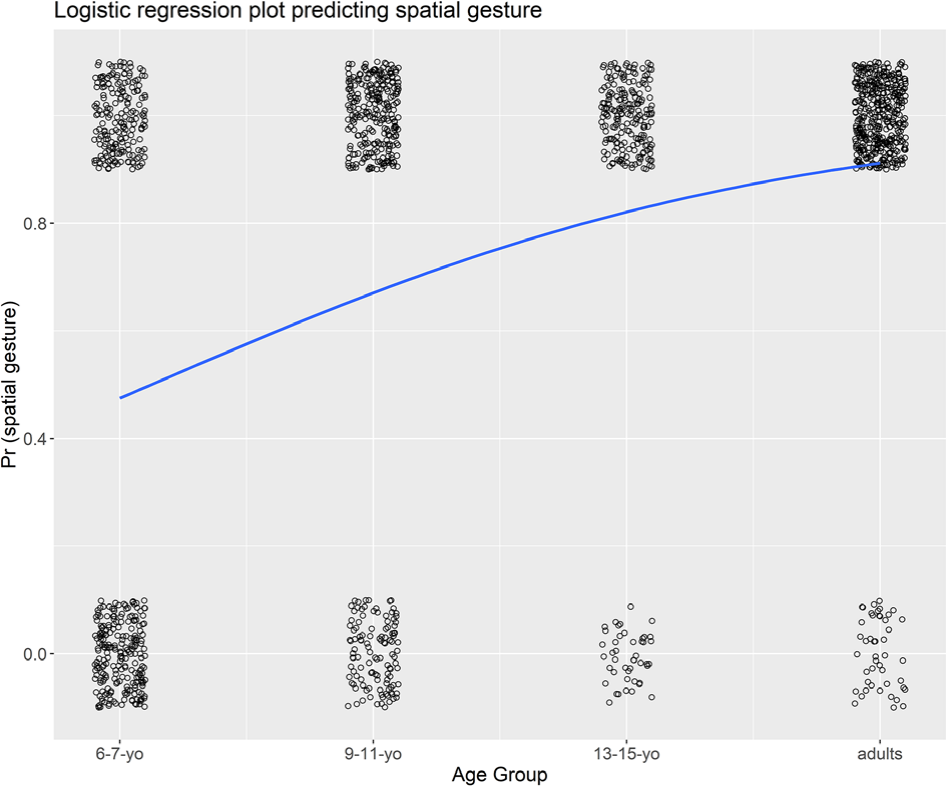
A logistic regression curve indicating the change in the probability that a spatial gesture was produced by age group. Individual data points for the data from each opportunity to produce a gesture are presented jittered; note that this is binary data (a spatial gesture was produced or not produced), so scores are either 0 or 1.

To test for the presence of a linear trend, age group was coded as a linear variable ranging from 0 (6‐to‐7‐year‐olds) to 3 (adults). The model intercept varied by both subject and item. The model, as presented in Table 1, indicates a significant positive linear trend (from youngest age group to oldest) in the likelihood that on any given opportunity for gesture production, a spatial gesture was produced. Fig. 1 displays the logistic regression curve for this model along with the jittered data points, 1 representing the presence of a spatial gesture and 0 representing the absence. We compared the model in Table 1 with a nested model in which the age group factor is removed. A chi‐square difference test indicated that removing the effect of age group from the model significantly reduced the model fit, χ^2^(1) = 47.50, *p* < .001. The full model also had a lower Akaike Information Criterion score (AIC full = 1,115) than the reduced model (AIC reduced = 1,160.5). Lower AIC scores indicate better model fit.

**Table 1:** Model predicting the likelihood of producing a spatial gesture

	β (*SE*)	95% CI	*Z*
Intercept	−0.56 (0.56)	−1.72–0.58	−1.01
Age group	1.44 (0.21)	1.04–1.88	6.85***

Note.

*** *p* < .001.

Finally, the proportion of children in each age group that produced at least one spatial gesture across the 12 possible opportunities was calculated. A majority of 6‐to‐7‐year‐olds produced at least one spatial gesture (69.2%). This figure rose to 87.8% for 9‐to‐11‐year‐olds and 100% for 13‐to‐15‐year‐olds and adults.

### 5.2 Spatial gestures by axis

We next examined the orientation of the spatial gestures participants made. Overall, 70.9% of spatial gestures were made in the lateral axis, 28.6% in the sagittal axis, and 0.5% in the vertical axis (see Table 2 for a breakdown by age group). As so few gestures were produced in the vertical axis, analysis focused on the relative proportion of lateral to sagittal gestures. We fitted an initial mixed effect model with a logit function predicting the probability that a lateral gesture was observed.^2^ Age group was a predictor in the model and was coded as a linear variable, while the intercept varied by subject and item. A chi‐square difference test comparing this model to a random intercepts model only (i.e., removing the age group term) indicated that the effect of age group did not significantly improve the model fit, χ^2^(1) = 0.99, *p* = .319 (AIC for model with age group factor = 561.4, AIC without age group factor = 560.3). Because age group was not straightforwardly linearly related to orientation (see Table 2), a second model with age group coded as a categorical variable (i.e., nonlinear) with four levels and the reference category set to the adult group was produced, and is presented in Table 3. Overall 57.6% of the spatial gestures adults produced were in the lateral axis. In comparison to adults, both the 6‐to‐7‐year‐old age group and the 13‐to‐15‐year‐old age group produced a greater proportion of lateral gestures relative to sagittal gestures (83.8% and 92.3%, respectively). The 9‐to‐11‐year‐olds, however, did not differ significantly from the adult group on the relative proportion of lateral to sagittal gestures (68%).

**Table 2:** For each age group, percentage of opportunities on which a spatial gesture was produced; percentage of these spatial gestures that were lateral, sagittal, or vertical; the percentage of lateral and sagittal gestures that were congruent; and the percentage of spatial gesture pairs that marked magnitude asymmetries

Age Group	Spatial Gestures	Orientation of Spatial Gestures			Congruency of Spatial Gestures		Magnitude Marked
		Lateral	Sagittal	Vertical	Lateral	Sagittal	
6‐to‐7	45.9	82.5	16	1.5	82.8	90.3	23.5
9‐to‐11	69.7	67.8	31.8	0.4	84.5	92.9	22.5
13‐to‐15	82.1	92.2	7.8	0	79.1	93.8	36.3
Adults	90.4	57.4	42.2	0.4	97.6	98.4	53.4

**Table 3:** Model predicting the likelihood of producing a lateral gesture with adults as the reference category

	β (*SE*)	95% CI	*Z*
Intercept	1.00 (1.48)	−1.91–3.91	0.67
6‐to‐7‐year‐olds	6.55 (1.88)	2.87–10.22	3.49***
9‐to‐11‐year‐olds	2.95 (2.53)	−2.00–7.90	1.17
13‐to‐15‐year‐olds	7.42 (2.00)	3.50–11.35	3.71***

Note.

*** *p* < .001.

### 5.3 Congruency of spatial gestures

The majority of gestures (89.6%) produced in the lateral and sagittal axis was congruent with canonical space–time mappings in an English‐speaking population (see Table 2 for a breakdown by age group and axis). We examined whether age group and axis affected the likelihood that a congruent gesture was observed. We began by fitting an initial model with age group (coded as a linear variable), axis (lateral vs. sagittal), and the interaction of age group and axis as fixed effects. Participant and item were included as random intercepts. The full model was compared to the nested model with the interaction term removed. The reduced model did not significantly lessen model fit and was retained, χ^2^(1) = 0.68, *p* = .411 (AIC for full model = 478.6, AIC for reduced model = 477.3). The reduced model was compared to two further nested models, one with the age group factor removed and one with the axis factor removed. Removing the age group factor significantly reduced the model fit, χ^2^(1) = 5.99, *p* = .014 (AIC for full model = 477.3, AIC for reduced model = 481.3). Removing the axis factor also reduced the model fit, χ^2^(1) = 4.36, *p* = .037 (AIC for full model = 477.3, AIC for reduced model = 479.7); therefore, both factors were retained. The equation for the final model is presented below and the model is summarized in Table 4.

$$\mathrm{Pr}\left(congruent\;gesture\right)=logit^{-1}\left(\beta_{0}+Subj_{0j}+Item_{0k}+\beta_{1}.Age\;group+\beta_{2}.Axis+\epsilon_{i}\right)$$

$$Subj_{0j}∼N\left(0,\;\sigma_{\mathrm{subj}}^{2}\right),\;\text{for}\;j=1,\;\ldots ,\;132$$

$$Item_{0k}∼N\left(0,\;\sigma_{\mathrm{item}}^{2}\right),\;\text{for}\;k=1,\;\ldots ,\;8$$

**Table 4:** Model predicting the likelihood of producing a congruent gesture with age group and axis as predictors

	β (*SE*)	95% CI	*Z*
Intercept	3.47 (0.82)	2.04 to 5.55	4.21***
Age group	0.79 (0.32)	0.17 to 1.47	2.48*
Axis	−0.84 (0.42)	−1.70 to −0.05	−2.03*

Note.

* *p* < .05

*** *p* < .001.

**Figure 2:**
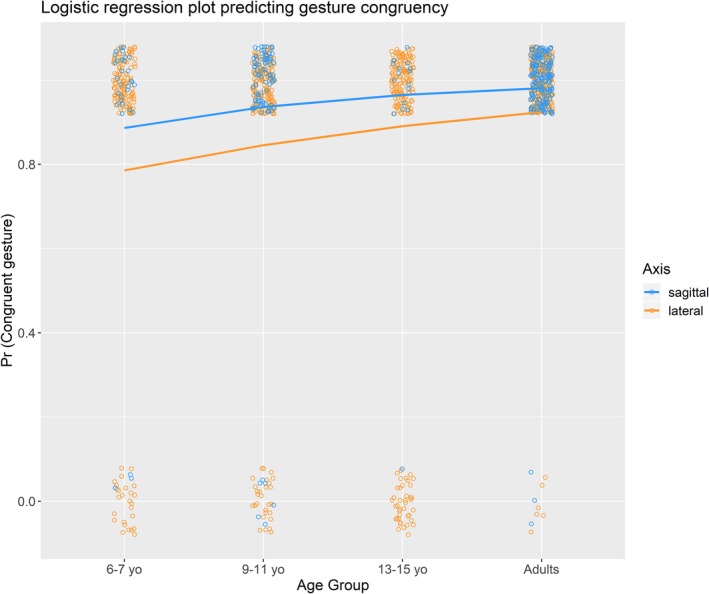
Logistic regression curves indicating the change in the probability that a congruent gesture is produced by age group and axis. Individual data points are presented jittered; note that these are binary data (gesture either congruent or incongruent) and therefore only scores of 0 and 1 are possible.

The model is displayed in Fig. 2 and indicates a positive linear effect of age group on the probability of producing a congruent gesture. There was also a significant effect of axis such that relative to sagittal gestures, lateral gestures are less likely to be congruent.

### 5.4 Marking of magnitude

Our final analysis considered whether individuals mark magnitude differences in time from the present in the production of spatial gestures. We considered only those pairs of temporal terms that were asymmetric in their temporal distance from the present and on which participants produced two spatial gestures. Of these pairs, 7.4%^3^ were instances in which the participant switched axis. The propensity to switch axis did not vary with age group, Wald χ^2^(3) = 3.04, *p* = .39. Due to the difficulty of directly comparing the magnitude of gestures produced in different axes, we only considered those pairs of gestures that were produced along the same axis. An initial model included age group (coded as a linear variable), axis (lateral vs sagittal), and the interaction of age group as fixed effects and subject and item pair as random intercepts. The full model was compared to a nested model with the interaction term removed. Model fit was not significantly reduced by removing the interaction term and so the reduced mode was retained, χ^2^(1) = 0.01, *p* = .907 (AIC for full model = 316.9, AIC for reduced model = 314.9). The reduced model was compared to two further nested models, one with the age group factor removed and one with the axis factor removed. Model fit was significantly reduced by removing the age group factor, χ^2^(1) = 12.84, *p* < .001 (AIC for model with age group term = 314.9, AIC for model without age group term = 325.7). Likewise, model fit was significantly reduced by removing the axis fit, χ^2^(1) = 6.29, *p* = .012 (AIC for model with axis term = 314.9, AIC for model without age group term = 319.2). The equation for the final model is presented below and the model is summarized in Table 5.

$$\mathrm{Pr}\left(magnitude\;marked\right)=logit^{-1}\left(\beta_{0}+Subj_{0j}+Item_{0k}+\beta_{1}.Age\;group+\beta_{2}.Axis+\epsilon_{i}\right)$$

$$Subj_{0j}∼N\left(0,\;\sigma_{\mathrm{subj}}^{2}\right),\;\text{for}\;j=1,\;\ldots ,\;133$$

$$Item_{0k}∼N\left(0,\;\sigma_{\mathrm{item}}^{2}\right),\;\text{for}\;k=1,\;\ldots ,\;4.$$

**Table 5:** Model predicting the likelihood of marking magnitude differences in their gesture with age group and axis as predictors

	β (*SE*)	95% CI	*Z*
Intercept	−2.34 (0.90)	−4.50 to −0.62	−2.62**
Age group	1.02 (0.33)	0.45 to 1.80	3.11**
Axis	−1.68 (0.71)	−3.27 to −0.36	−2.36*

Note.

* *p* < .05

** *p* < .01.

**Figure 3:**
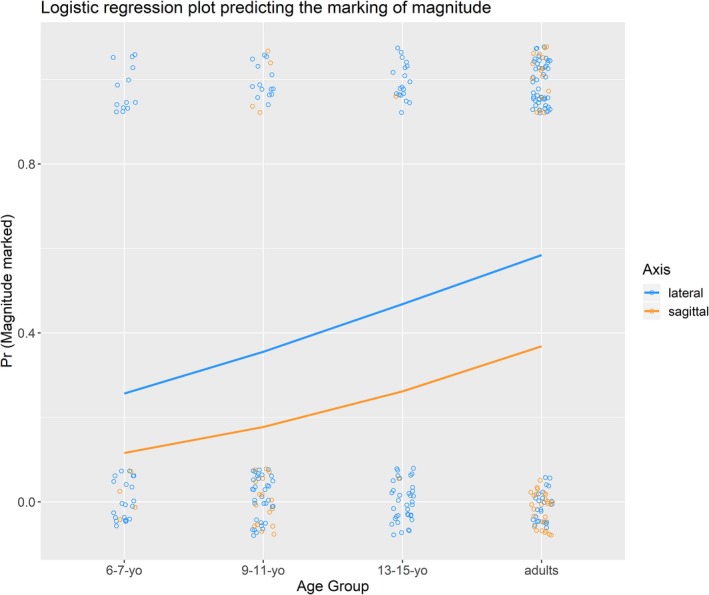
Logistic regression curves predicting the likelihood that magnitude differences are marked as a function of age group and axis. Individual data points are presented jittered; note that these are binary data (gesture either marked for magnitude or not marked) and therefore only scores of 0 and 1 are possible.

The model, as displayed in Fig. 3, indicates a positive linear effect of age group on the probability that magnitude differences are marked in gestures (overall adults marked magnitude differences on 66.2% of opportunities whereas that figure reduced to 38.2% for 6‐to‐7‐year‐olds). In addition, the model indicates that magnitude differences are more likely to be marked in the lateral axis than the sagittal axis (44.7% of lateral gesture pairs marked magnitude compared to 29.4% of sagittal pairs).

## 6 Discussion

As far as we are aware, this study is the first to examine in detail the developmental profile of the spatial gestures that are produced when talking about time. We found that children from at least 6 years of age onwards produce spatial metaphorical gestures when talking about time. However, the propensity to do so increases with age, with adults producing around twice as many such gestures as 6‐to‐7‐year‐olds. When we examined the axes along which participants gestured, we found a mixed pattern. In comparison to the adults, both the 6‐to‐7‐year‐olds and adolescents produced fewer sagittal relative to lateral gestures. The 9‐to‐11‐year‐olds, however, were somewhere intermediate between the adults and the younger children and adolescents: Although a higher proportion of their gestures than those of adults were lateral rather than sagittal (68% compared to 58%), these proportions were not significantly different. When we examined whether the gestures participants made in the lateral and sagittal planes were congruent with canonical space–time mapping for English speakers, two patterns emerged. First, although the majority of their gestures were congruent, children and adolescents made significantly more incongruent mappings than adults. Second, across the sample as a whole, incongruent mappings were more common among lateral gestures than sagittal gestures. Finally, we found that the tendency to mark temporal distance from present in gesture was influenced by both age group and the axis along which the gesture was performed, with more magnitude marking on the lateral plane and more marking in older participants.

### 6.1 Language and gesture

What do the present data tell us about the relevance of studying co‐speech gestures? Consistent with previous studies, our data reveal important discontinuities between gesture and speech. As such, they highlight the importance of studying co‐speech gestures in their own right. In this study, we find children and adults alike spatialize time in their gestures while often describing time with literal rather than metaphorical language (e.g., “last week was seven days in the past”). On other occasions, we see participants of all ages employ sagittal spatial metaphors (e.g., “tomorrow is one day ahead of us”) while simultaneously gesturing in the lateral plane. Participants also switched axes mid‐explanation around 7% of the time, consistent with the idea that more than one spatial metaphor can be simultaneously active (Walker & Cooperrider, 2016). Indeed, lateral gestures for time, for which there are no corresponding linguistic metaphors in English, predominate in this study at all ages.

The use of different metaphors in spoken language compared to gestures raises interesting questions. On the one hand, that people produce metaphorical spatial gestures for time in the first place puts pressure on the contention that the spatial metaphors for time that are employed during everyday speech acts are “dead” metaphors, that is, conventionalized phrases devoid of metaphorical content. On the other hand, it is possible that people sometimes use contrasting metaphors in gesture and speech acts not because two different metaphors are simultaneously in conscious awareness but because they are not always alive to the metaphoricity in one or the other domain. Of course, much of the time when people gesture they are not aware of doing so (Goldin‐Meadow, 1999). For this reason, previous studies have regarded gesture analysis as a means of assessing implicit space–time mappings (Casasanto & Jasmin, 2012; Cooperrider & Núñez, 2009). In this study, however, we employed an elicited gesture paradigm, in which participants were explicitly told to use their hands. Casasanto and Jasmin argued that drawing speakers' attention to how they use their hands during talk about time encourages them to activate and use the sagittal space–time mapping established in language. Certainly, adults' use of sagittal gestures was more frequent (42% of spatialized gestures) in our study than has been reported in previous studies in which gestures were not deliberately elicited (see Casasanto & Jasmin, 2012, Study 2; Walker & Cooperrider, 2016, Study 2), though not as frequent as sagittal gestures in Casasanto and Jasmin's (Study 1) study of elicited gesture (58.6% of gestures were sagittal). Nevertheless, we found that lateral gestures predominated, particularly among the 6‐to‐7‐year‐olds and the adolescents. We note, though, that it may be that posture is also relevant to the axis in which participants gesture. Our participants were seated throughout (as were participants in Casasanto & Jasmin and Walker & Cooperrider). It may be that participants when standing have more freedom for movement in the sagittal plane, and this may be worth examining in future studies.

Although the relative proportion of lateral to sagittal gestures differed greatly between the youngest age group and adults (Table 2), the performance of the two intermediate age groups make drawing firm conclusions about the overall pattern of developmental change between childhood and adulthood difficult. Although the 9‐to‐11‐year‐olds also produced relatively fewer gestures that were sagittal versus lateral than the adults, this difference was not significant, whereas the difference was significant between the 6‐to‐7‐year‐olds and adults. In the absence of the data from the adolescent group (see Table 2), one might have been tempted to conclude that the proportion of sagittal gestures increases across childhood and into adulthood. However, if this was the case, we would have expected the adolescent sample to be even more similar to the adult group than the 9‐to‐11‐year‐olds; in fact, the adolescents differed significantly from the adults and were actually more similar to the youngest children in producing relatively few sagittal gestures. If there is an anomaly here, it is not possible to tell whether it lies with the performance of the adolescents (producing fewer sagittal gestures than one might expect) or the 9‐to‐11‐year‐olds (producing more sagittal gestures than one might expect). Future developmental studies might usefully focus on later childhood and adolescence in order to provide a clearer and more complete picture of developmental change.

### 6.2 Interpreting the developmental findings

The developmental findings run contrary to one possible prediction based on the existing literature that was discussed in the introduction, that is, that sagittal gestures might be expected to be more common in younger children than adults. That so few sagittal gestures relative to lateral gestures are produced by the youngest age group (16%) suggests that linguistic metaphors do not play an important role in the acquisition of space–time mappings, given that English employs sagittal but not lateral space–time metaphors. Rather, the predominance of lateral gestures at this age suggests that culturally determined conventions such as reading and writing direction and pictorial displays of time or of the number line are more important in establishing space–time mappings. One consideration here is the age with which children in the UK education system begin formal instruction in literacy and numeracy, which is early by international standards, at around 4–5 years. Thus, 6‐to‐7‐year‐olds in this study will typically have had at least 2 years' explicit instruction in reading and writing.

Cross‐cultural evidence for an influence of reading and writing culture on space–time mappings in children has previously been reported by Tversky et al. (1991). Tversky and colleagues asked Arabic‐, English‐, and Hebrew‐speaking children to place stickers representing events on a blank sheet of paper. Arabic children, whose script is written right to left, predominantly arranged temporal sequences in a right‐to‐left fashion. English‐speaking children predominantly arranged temporal sequences in a left‐to‐right fashion, whereas Hebrew‐speaking children were intermediate between the two. More recently, using a similar technique, Tillman et al. (2018) reported that although English‐speaking North American pre‐schoolers did not show a preference for the conventional left–right time–space mapping for sequences, when children started formal education around 5–6 years, they began to spontaneously prefer this mapping over either right‐left or vertical mapping. Although these findings of studies using card arrangement tasks suggest that cultural conventions guide time–space mappings once children start school, they cannot inform us about the relative tendency to map time onto the lateral versus sagittal axis: Children in these studies were arranging sequences on a two‐dimensional space that only allowed for vertical or horizontal mappings. This study allowed for observation of spontaneous use of the sagittal axis, with the findings indicating that children do not preferentially make use of this axis.

As mentioned in the Introduction, some authors have argued that the sagittal space–time mapping has privileged status due to the way people navigate the environment (Rinaldi et al., 2016, 2018). In adults, reaction time data suggest that representations of time in the sagittal axis, as measured by space‐time congruency effects, are stronger than those in the lateral axis (Eikmeier, Alex‐Ruf, Maienborn, & Ulrich, 2015), and English speakers tend to lean forward when thinking about the future and backward when thinking about the past (Miles, Nind, & Macrae, 2010). By contrast, an interpretation of the present data as an illustration of how culturally determined conventions influence 6‐to‐7‐year‐olds' space–time mapping is consistent with findings indicating that experience with written text is the critical determinant (Bottini, Crepaldi, Casasanto, Crollen, & Collignon, 2015; Casasanto & Bottini, 2010). These studies suggest that visuo‐motor and attentional motor effects are as important as locomotor effects in establishing space–time mappings (see also Ouellet, Santiago, Israeli, & Gabay, 2010, for cross‐cultural effects of writing direction).

The findings of Tillman et al.'s (2018) card arrangement study, which used younger children (4‐to‐5‐year‐olds) than in the current study, indicated that time–space mappings are initially very malleable (as demonstrated by priming effects) before becoming highly consistent in adulthood with conventional mappings. Studies such as that of Casasanto and Bottini (2010), in which space‐time congruency effects in the lateral plane are reversed after reading mirror‐reversed script, suggest that spatial representations of time can be labile even in adults (see also Li & Cao, 2018, for an example of the lability of the sagittal plane). Nevertheless, in the absence of experimental manipulation, as in the current study, adults will produce gestures that are almost always congruent with canonical time–space mappings. Children, and even adolescents, showed less consistency than adults in the adherence to the canonical mappings in both the lateral and sagittal axis (see Fig. 2). This suggests that space–time mappings are not as strongly established in children as they are in adults.

In fact, inspection of the distribution of incongruent mappings across participants further indicates that incongruent mappings were strongly bimodal, with the overwhelming majority of participants consistently mapping time on to space congruently and a small number of participants who predominantly engaged in the reverse mapping. This suggests that a proportion of the younger participants were still getting to grips with the canonical mappings. Across all participants, gestures in the lateral axis were more likely to be mapped incongruently than those in the sagittal axis. One reason for this likely arises from the asymmetry of the human body along the sagittal axis compared with the symmetry of the body along the lateral axis. The asymmetry makes it relatively easier to map the early to late polarity of time consistently. Indeed, the asymmetry of the human body along the sagittal axis is regularly cited as a reason for why time is preferentially lexicalized on this axis across languages (Radden, 2004).

The final developmental finding to be considered is the age‐related change in the likelihood that participants marked the relative magnitude of distances of temporal locations from the present. Disentangling the precise effect that age has on marking magnitude is difficult because there were age group differences in the relative proportion of lateral to sagittal gestures, with magnitude being more likely to be marked in the former than the latter axis. That there is a main effect of orientation on magnitude marking is unsurprising given the physical difficulties of marking magnitude when gesturing backwards. Indeed, there were only three observations of participants marking magnitude in the sagittal plane when the temporal terms were of different tense. If we limit our analysis to just instances where participants produced two spatial gestures in the lateral axis, we see a difference between the adults and the three younger age groups, with adults more likely to mark magnitude differences in their gestures than each of the children groups. Tillman, Marghetis, Barner, and Srinivasan (2017) have demonstrated that while 6‐year‐olds rarely make errors about the deictic status of the sort of time words used in the current study, knowledge about the relative remoteness of temporal locations improves considerably between 6 and 8 years. This suggests that the youngest children may have marked magnitude less than adults because they had a less firm grasp on the relative magnitudes of the distances.

Although this explanation may be plausible regarding young children's relative lack of magnitude marking, it does not explain why adults were around twice as likely as adolescents to mark magnitude, even though Tillman et al.'s (2017) findings suggest that 8‐year‐olds' judgments of the relative remoteness of temporal locations resemble those of adults. One possibility is that adults have simply finessed their use of gesture to incorporate remoteness as well as tense. An alternative is that adolescent spatial representations of time are continuing to mature. Intriguingly, Friedman (1989) has argued that, with regard to calendar time, there is a shift from early to mid‐adolescence in representational format. His claim is over the course of adolescence there is a shift from list‐like representations of days of the week and months of the year to spatialized representations, allowing greater flexibility and accuracy in use of the calendar. This accords with adolescents' own self‐reports of using mental images to think about calendar time. This suggests the possibility that the emergence of increasingly spatialized representations of calendar time supports the marking of magnitude in spatial gestures for time, although clearly this remains a speculative suggestion in the absence of further data. Given the current interactive technology, that children now typically have extensive access to, one potential source of influence for the increased spatialization of time in development is the use of devices such as tablets and smart phones. Many applications require users to swipe right and left for earlier than and later than content, for example, photo applications, scroll bars on video content. Again, while this seems a plausible factor impacting on the spatialization of time, we do not yet know the extent to which such experiences affect the developmental emergence of time–space mapping.

## 7 Summary and conclusion

This study demonstrates that children as young as 6–7 years will use spatial gestures that map time on to space, and that they typically do so in canonical ways. However, there is a developmental increase in the likelihood that such gestures are produced, and 6‐ to 7‐year‐olds are less likely than adults to use gestures on the sagittal axis. The gestures of children are also less likely to be canonical and to mark magnitude than those of adults. Taken as a whole, the findings suggest that although time–space mappings can be observed early in children's gestures, there are further improvements in gesture use as children become more familiar with conventional time–space mappings and time systems. The relative dominance of lateral compared to sagittal gestures in all groups of children means that this study provides no evidence to support either the idea that time–space mappings derive initially from linguistic metaphors, or that there is a privileged mapping of time on to the sagittal axis grounded in sensorimotor experience.

## Notes

1 An alternative explanation for the shift from lateral to sagittal gesturing was brought to our attention by an anonymous reviewer who notes that in the implicit gesture task, participants were given written instructions, whereas in the explicit gesture task, participants were given oral instructions. As we discuss later, spatial representations of time are influenced by reading behavior. For example, in an implicit association task, Hebrew speakers (subject to a right‐to‐left orthography) associated the right side of space with the past and the left side of space with the future, the opposite pattern to that seen in English and Spanish speakers (Ouellet, Santiago, Israeli, & Gabay, 2010). Moreover, even a brief exposure to mirror reversed orthography in Dutch speakers produced reaction times consistent with a right‐to‐left organization of earlier to later (Casasanto & Bottini, 2010).

2 Pr(sagittal gesture) = 1 − Pr(lateral gesture).

3 The figure was 6.7% when temporal term pairs that were equidistant from the present were included.
